# Lactic acid bacteria isolated from Kazakh traditional fermented milk products affect the fermentation characteristics and sensory qualities of yogurt

**DOI:** 10.1002/fsn3.2755

**Published:** 2022-04-19

**Authors:** Hui Li, Jiaxing Gao, Wenbo Chen, Chengjing Qian, Yong Wang, Jing Wang, Lishui Chen

**Affiliations:** ^1^ China‐Australia Joint Research Center for Dairy Future Technology Beijing Key Laboratory of Nutrition, Health & Food Safety Beijing Engineering Laboratory for Geriatric Nutrition Food Research COFCO Nutrition & Health Research Institute Beijing China; ^2^ 2541 Department of Chemical Engineering Monash University Clayton Victoria Australia; ^3^ Beijing Advanced Innovation Center for Food Nutrition and Human Health Beijing Technology & Business University Beijing China

**Keywords:** bacterial identification, lactic acid bacteria, starter culture, yogurt sensory qualities

## Abstract

Lactic acid bacteria (LAB) play a crucial role in the development of the taste, texture, and aroma of traditional fermented milk products. Five LABs from Kazakh traditionally prepared dairy products showed continuous subculture stability, as well as proper acidification and coagulation ability. They were identified as *Pediococcus pentosaceus* (1–5, 1–7), *Enterococcus faecium* (1–19), and *Lactobacillus plantarum* (1–12, 1–15). Their coagulation time and acidity values ranged from 5.97 to 12.78 h and 76.47 to 89.39°T. Yogurts prepared with *L. plantarum* were more condensed and textural integrity than those with *P. pentosaceus* and *E. faecium*. Determination of the volatile compound profiles suggested a higher diversity of volatile compounds than the control. The sensory evaluation presented positive overall sensory quality scores for the yogurts prepared with 1–12 and 1–15. The results provide additional information regarding the contributions of native LABs to the unique flavor and sensory qualities of traditionally prepared milk products. They may help to select starters or adjunct starters for developing distinctive, traditional nomadic fermented milk to satisfy consumer demand and increase market acceptability.

## Graphical abstract

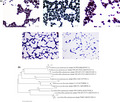

## 1 INTRODUCTION

The food in each region or area has particular characteristics because of the uniqueness of local ingredients and production techniques, which are deeply rooted in tradition and linked to the territory the food is derived from (Guarcello et al., 2016). The Kazakh herdsmen living in the Xinjiang Tianshan and Altai Mountain regions of northwest China have developed a variety of distinctive traditional, local fermented dairy products, such as milk curd, milk knots, yogurt, and vrum (the coagulated layer of fat on the surface of boiled milk which is dried by hanging it in a well‐ventilated place). These products are rich in nutrients, typically recognizable by their unique taste, and are popular in local markets (Zuo et al., 2014).

Lactic acid bacteria (LAB) play a crucial role in the development of the taste, texture, and aroma of traditional fermented milk products. They are widely distributed in nature and are well‐known as starters for traditional fermented products (Bao et al., 2011). Multiple researchers have examined the LAB species composition of traditional fermented products and expanded their potential industrial application. Ren et al. (2017) demonstrated that *Lactobacillus* was the most commonly isolated LAB species from fermented yak milk in central Tibet. *Enterococcus faecium* was detected in all wooden vats used for the production of traditional stretched cheeses in Italy (Scatassa et al., 2015). Recently, several studies have also explored the contribution of relevant strains of native LAB to the sensory properties of traditional dairy products (Choobari et al., 2021; Guarrasi et al., 2017; Madhubasani et al., 2020; Medeiros et al., 2016; Tian, Shi, et al., 2019; Tian, Xu et al., 2019).

To the best of our knowledge, limited work has focused on the contribution of LAB to the organoleptic properties of traditional Kazakh dairy products. Therefore, LAB strains in Kazakh dairy products were isolated, identified, and their fermentation characteristics were evaluated with the aim of providing additional information to select starters or adjunct starters for developing distinctive traditional fermented milk products such as yogurt or cheese.

## 2 MATERIALS AND METHODS

### 2.1 Sampling

Five traditionally prepared samples (one cheese, one milk knot, two yogurts, and one vrum) were obtained from local Kazakh herding families living in Tianshan Mountain, Xinjiang, China. The samples were collected aseptically in sterile bags, kept in an ice‐box container during transit to the laboratory, and stored at −20°C until analysis.

### 2.2 LAB isolation

Samples were isolated using two selective media, MRS and M17 (Beijing Land Bridge Technology), under both aerobic and anaerobic conditions at 37 and 30°C for 72 h to obtain as many LAB strains as possible. Isolates were selected and purified according to previously published methods (Medeiros et al., 2016).

All isolates were verified as LAB using a combination of Gram reaction, catalase activity, and morphological analysis. Gram reactions were visualized using an optical microscope (Scope A1; Carl Zeiss Microscopy) under oil immersion at 100‐fold magnification. Colony morphology was recorded using a colony counter Scan1200 (Interscience International). Cocci, bacilli, or coccobacilli colonies that were gram‐positive with a negative catalase result were included in the LAB group (Abosereh et al., 2016). Isolates were stored at −20°C in MRS broth supplemented with 20% (v/v) glycerol and only activated prior to testing by two sequential transfers in the same broth used in the experiments.

### 2.3 Primary selection of LAB with milk fermentative characteristics

The microbial culture was inoculated at a level of 2 ml/100 ml in sterile reconstituted skim milk (120 g/L) (BD Biosciences Pharmingen) fortified with 6% sucrose and incubated at 42°C. Each strain was subcultured 10 times in sterile reconstituted skim milk. After 10 generations, the strains with continuous subculture stability, suitable acidification, and coagulation ability were selected for further analysis.

### 2.4 LAB identification

Strains were harvested for DNA extraction and purification after cultivation in MRS broth at 37°C for 18 h. Total DNA was extracted using a Bacterial Genomic DNA Extraction Kit (Tiangen). DNA concentration and quality were determined with a NanoDrop 2000 ultra‐microspectrophotometer (Thermo Fisher Scientific). The 16S rRNA gene sequence primers used for PCR amplification were 27F (5′‐ AGA GTT TGA TCC TGG CTC AG ‐3′) and 1492R (5′‐TAC GYT ACC TTG TTA CGA CTT ‐3′) (Rashid & Hassanshahian, 2014). Amplification was performed in a Veriti 96‐well thermal cycler (Life Technologies). The PCR temperature profile was as follows: 95°C pre‐denaturation for 10 min, 35 cycles of 95°C denaturation for 30 s, 58°C annealing for 30 s, and 72°C extension for 30 s, then a 72°C extension for 10 min. PCR products were sequenced by Invitrogen Shanghai Trading Co. Ltd.

The 16S rRNA gene nucleotide sequences of the five isolates were analyzed and identified using the BLAST program on the NCBI website (http//:www.ncbi.nlm.nih.gov/blast). Alignments were performed to construct a phylogenetic tree and compare similarities among the sequences using the neighbor‐joining method in MEGA software version 6.0 (http://www.megasoftware.net) and bootstrapped with 1000 replicates (Tamura et al., 2013).

### 2.5 Fermentation characterization of selected strains in yogurt manufacturing

Yogurt was made from whole milk (Sanyuan Dairy). The milk was homogenized, pasteurized at 95°C for 15 min, and cooled to 40°C before the selected microbial culture was inoculated (2 ml/100 ml). Samples were incubated at 42°C until coagulation (until pH reached 4.6). The samples were immediately cooled in an ice‐water bath and stored at 4°C for 12 h to mature and the acidity was recorded. Texture, flavor compounds, and sensory qualities were analyzed. *Lactobacillus delbrueckii* subsp. *bulgaricus* CGMCC 1.1480, (China General Microbiological Culture Collection Center, Beijing, China) preserved at the China‐Australia Joint Research Center for Dairy Future Technology was used as the control.

### 2.6 Acidification and coagulation analysis

The pH was measured using a PB‐10 pH meter (Sartorius Scientific Instruments). Acidity was determined by titration with 0.1 N NaOH using phenolphthalein as an indicator, and the results were expressed in Thorner degrees (°T). Coagulation activity was defined as clotting after incubation at 42°C within 4–6 h (fast), 6–12 h (medium), or >12 h (slow) time frames (Ayad et al., 2004; Yi et al., 2011).

### 2.7 Textural analysis

Yogurt texture was evaluated by the backward‐extrusion test using a TA‐XT Plus texture analyzer (Stable Micro Systems) equipped with a 5 kg loading cell (Mousavi et al., 2019; Wang et al., 2019). The parameters were modified from the Exponent template: 35 mm diameter cylinder probe, 1.0 mm/s test speed, 30 mm penetration distance, and 10 g surface trigger force. The tests were carried out on samples prepared in 125 ml containers (64 mm diameter and 70 mm height). Hardness (g), springness (%), consistency (g × s), and gumminess index (g) were calculated using the Exponent program, based on the instruction manual supplied by the manufacturer.

### 2.8 Volatile compound analysis

Twenty grams of yogurt was weighed and mixed with 5 g NaCl in a 60 ml glass vial. Five grams of the mixture was placed in a 20 ml head‐space vial sealed with a polytetrafluoroethylene‐faced silicone septum (VWR). Extractions were carried out with a solid phase microextraction device (Supelco) containing a fused‐silica fiber coated with a 50/30 μm layer of Divinylbenzene/Carboxen/Polydimethylsiloxane. The vial was equilibrated at 60°C, centrifuged at 300 rpm for 2 min, and the fiber was exposed to the headspace over the sample for 30 min. The fiber was conditioned before adsorption by heating it in the gas chromatograph injection port at 250°C. After adsorption, the fiber was removed from the vial and immediately inserted into the GC‐MS injection port (Bezerra et al., 2017).

Separation was performed on an Agilent 7890A gas chromatograph (Agilent Technologies) coupled to a mass detector (Agilent 5975C) and DB‐WAX capillary column (60 m × 250 μm × 0.25 μm). The temperature program was as follows: 3 min at 32°C, 12°C/min ramp to 48°C, hold for 10 min, 6°C/min ramp to 130°C, 10°C/min ramp to 200°C, 20°C/min ramp to 230°C, and hold for 2 min. The injection port temperature was 250°C, and the flow rate was 1.0 ml/min. The mass spectrometer was operated in electron impact mode with a source temperature of 230°C, an ionization voltage of 70 eV, and a scan range from 29 to 400 *m*/*z* at 3.33 scans/s. Identification of the volatile compounds was based on comparison of their mass spectra with those from previously analyzed authentic compounds (spectra from the 14.0 NIST spectrum library). The retention index used a homologous series of alkanes (C6‐C28; Sigma‐Aldrich). To quantify the volatile compounds, 2‐octanol was used as an internal standard. Results were represented as the percentage of each compound, thus allowing strain comparisons based on the relative contents of each compound, but not of their concentrations in sample cultures (Cheng et al., 2017; Guarrasi et al., 2017).

### 2.9 Sensory evaluation

According to Ao et al. (2012) and Hashim et al. (2009), 12 panelists aged 23–46 y (staff from the China‐Australia Joint Research Center for Dairy Future Technology selected based on interest and experience in the sensory evaluation of yogurt and fermented milk) performed the sensory evaluation of the yogurts. Panelists assessed the fermented yogurt characteristics using a 10‐point scale based on the following attributes: appearance (1 = no curd, 10 = smooth yogurt gel without whey), flavor (1 = yogurt with unsatisfactory odor, 10 = yogurt with satisfactory, fermented flavor), taste (1 = weak acidity or over acidity, 10 = suitable acidity), and overall quality (1 = poor quality, 10 = excellent quality).

### 2.10 Statistical analysis

All experiments were performed in triplicate and results were expressed as the mean ± SD. Statistical analyses were performed by IBM SPSS Statistics 20 (IBM Inc.). One‐way analysis of variance was used to compare the means. Mean separations were performed by S‐N‐K (Newman–Keuls). Differences at *p* < .05 were considered significant. Additionally, principal component analysis (PCA) was performed with JMP Pro 16 software.

## 3 RESULTS AND DISCUSSION

### 3.1 Isolation and selection of the fermentative LAB

Nineteen LAB strains were selected from MRS and M17 plates according to morphological analysis of colony color, shape, and gloss. Of these, 14 strains were identified as gram‐positive and catalase‐negative cocci; the other five were gram‐positive and catalase‐negative bacilli presumed to be LAB.

Five strains with continuous subculture stability, proper acidification, and coagulation ability were selected for further analysis as these qualities are essential for producing high‐quality constant fermentation dairy products (Ayad et al., 2004). The five strains were coded as 1–5, 1–7, 1–12, 1–15, and 1–19.

**FIGURE 1:**
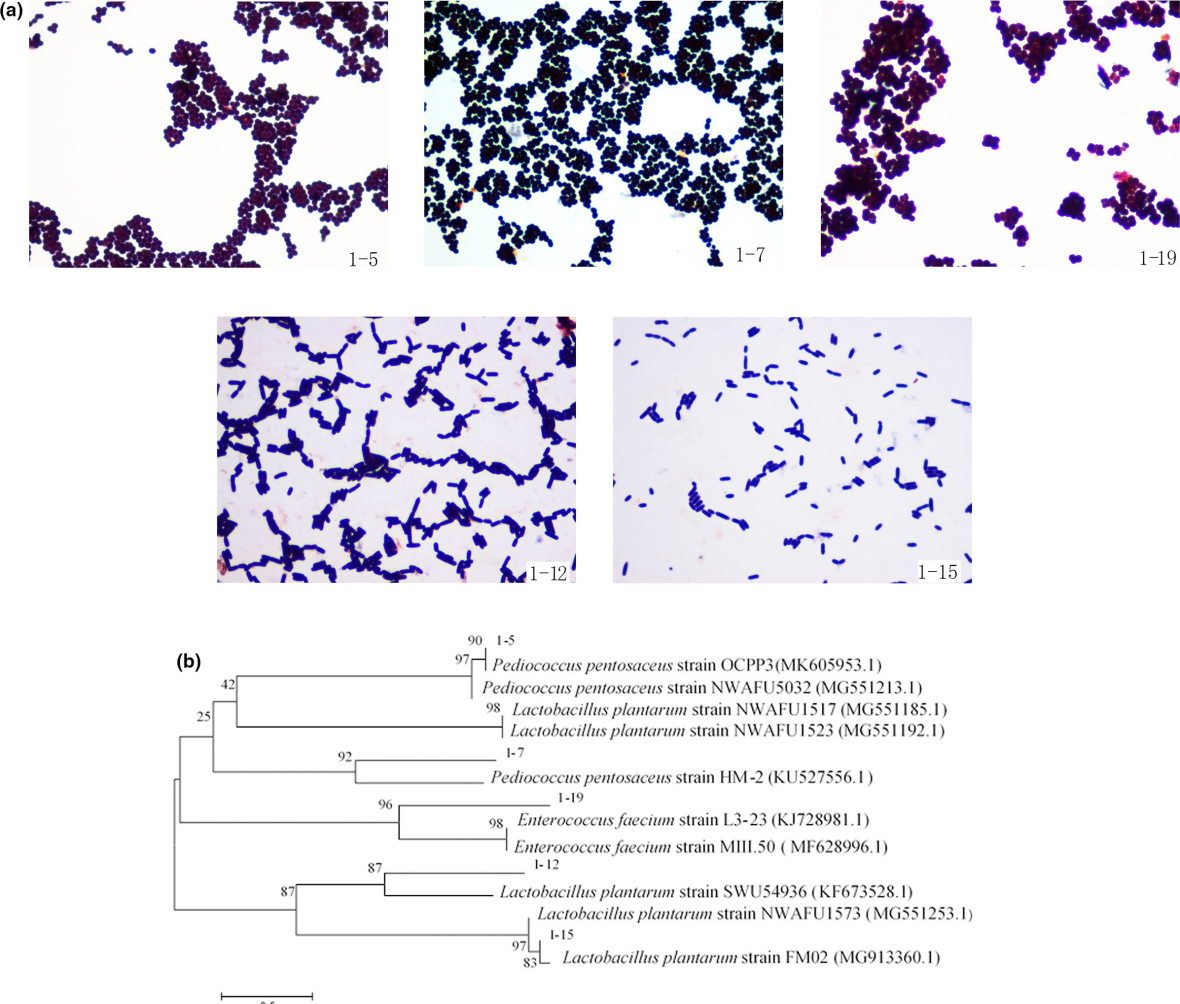
(a) Strain evaluation by Gram staining of the five selected strains (×1000), and (b) phylogenetic tree analysis based on 16S rRNA gene sequences of the five selected isolates

Gram stain results showed that strains 1–5, 1–7, and 1–19 were gram‐positive cocci, while strains 1–12 and 1–15 were gram‐positive and rod‐shaped (Figure 1a). Additionally, molecular identification suggested that the five strains were *Pediococcus pentosaceus* (1–5, 1–7), *Enterococcus faecium* (1–19), and *Lactobacillus plantarum* (1–12, 1–15). Phylogenetic tree evaluation confirmed the molecular identification results (Figure 1b).

### 3.2 Fermentation time and acidity assays of the five selected strains

**FIGURE 2:**
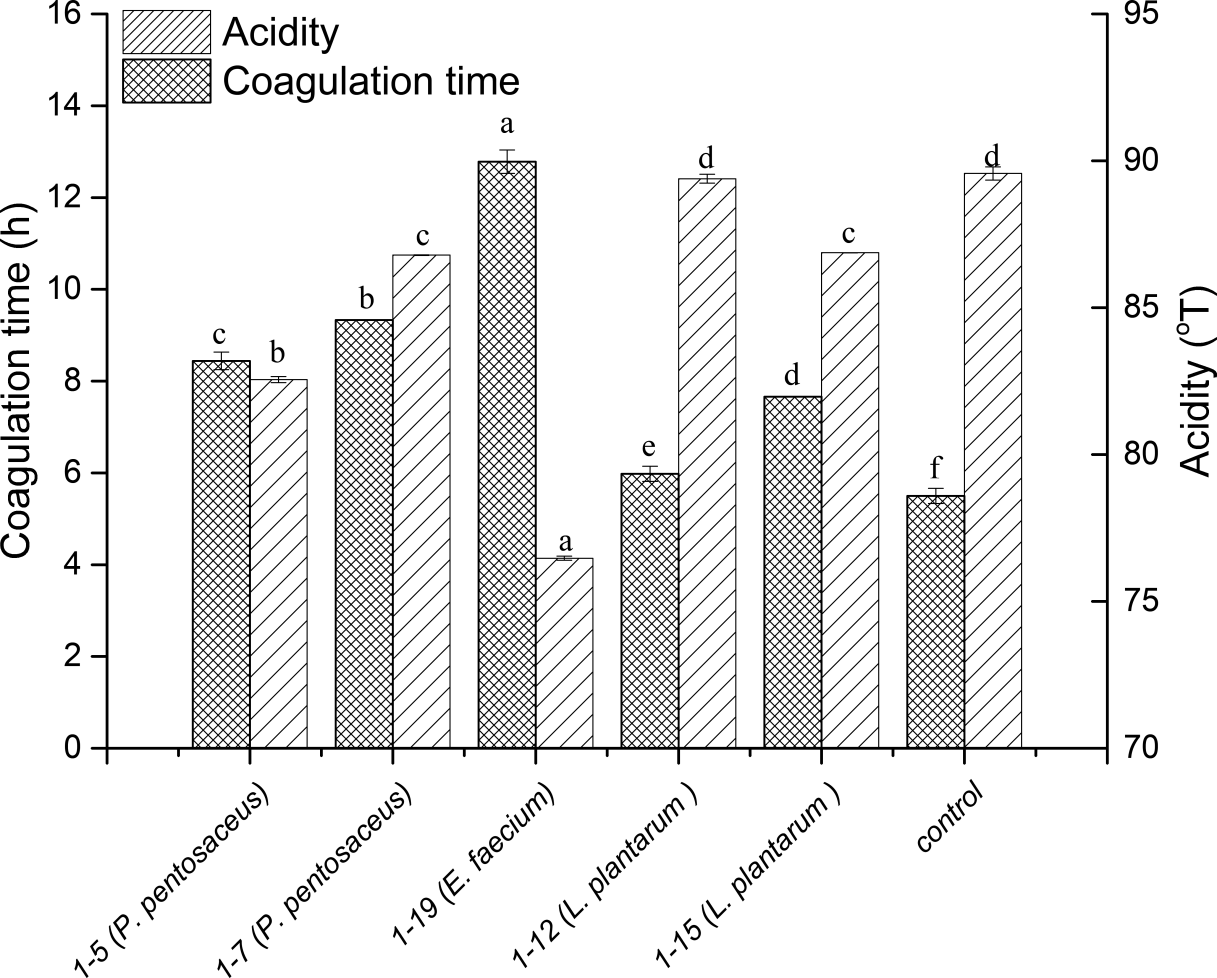
Fermentation time and acidification of the selected LAB isolates. Values are expressed as the mean ± SD. a–f: Different letters above the same column indicate significant differences (*p < .05)

Strain 1–12 (*Lactobacillus plantarum*) produced higher levels of acid (89.39°T) and had a lower clotting time (5.97 h) than the other strains, followed by strain 1–15 (*Lactobacillus plantarum*) (86.87 °T; 7.66 hr) (Figure 2). Previous reports showed that *Lactobacilli* have fast or medium acidification and coagulation activity (Yi et al., 2011). Ayad et al. (2004) demonstrated that most *L. plantarum* strains are active acid‐forming agents and coagulate milk in 3–9 h. Despite having medium coagulation activity (clotting time from 8.44–12.78 h), strains 1–7 (*Pediococcus pentosaceus*), 1–5 (*Pediococcus pentosaceus*), and 1–19 (*Enterococcus faecium*) generated high acidity levels (>70.00°T), indicating that these strains were good candidate starters for the dairy fermentation process.

### 3.3 Yogurt textural properties

Textural characteristics of the yogurts are listed in Table 1. Strain 1–12 showed the highest hardness (187.48 g), springness (69.10%), and consistency (3804.66 g × s) values, which were similar to those of the control, followed by those of strain 1–15, which also showed high hardness, springness, and consistency values. Strains 1–12, 1–15, and 1–7 showed similar gumminess values to that of the control (19.38 g). According to Cheng et al. (2017), texture is an essential aspect of yogurt quality and plays an important role in sensory evaluation and consumer acceptability. Yogurts fermented by strains 1–12 and 1–15 were condensed and had integrity, suggesting their potential in fermented dairy product preparation.

**TABLE 1:** Textural parameters of yogurts fermented by the five selected strains

Species	Strain	Hardness (g)	Springness (%)	Consistency (g × s)	Gumminess (g)
*Pediococcus pentosaceus*	1–5	122.71 ± 2.01^b^	51.17 ± 1.44^b^	3396.43 ± 2.18^b^	16.33 ± 0.44^b^
*Pediococcus pentosaceus*	1–7	153.44 ± 1.00^c^	57.59 ± 1.51^c^	3657.48 ± 3.40^c^	20.30 ± 0.97^c^
*Enterococcus faecium*	1–19	107.81 ± 2.28^a^	45.76 ± 1.23^a^	2083.33 ± 3.22^a^	13.41 ± 1.28^a^
*Lactobacillus plantarum*	1–12	187.48 ± 2.25^e^	69.10 ± 1.67^d^	3804.66 ± 2.08^e^	19.45 ± 0.69^c^
*Lactobacillus plantarum*	1–15	181.47 ± 0.93^d^	68.80 ± 2.59^d^	3772.41 ± 1.18^d^	20.01 ± 0.99^c^
*Lactobacillus delbrueckii* subsp. *Bulgaricus*	Control strain	186.59 ± 1.26^e^	67.78 ± 1.23^d^	3801.39 ± 1.84^e^	19.38 ± 0.83^c^

Note

Values are expressed as mean ± SD. Different letters within the same column indicate significant differences (*p < .05).

### 3.4 Volatile compound profile

Determination of the volatile compound profiles suggested that the five strains produced a higher diversity of volatile compounds than the control (Table 2). Thirty‐six volatile compounds produced by the five sample strains were detected and grouped into seven chemical categories: ketones, alcohols, aldehydes, acids, ethers, esters, and polyaromatic hydrocarbons.

**TABLE 2:** Volatile compounds of each yogurt sample analyzed by SPME‐GC‐MS

	Volatile components	RI	RI L	Relative contents (%)					
		WAX	WAX	1–5	1–7	1–19	1–12	1–15	Control
Ketones	2,3‐butanedione	1043	1056	8.50 ± 0.03	7.86 ± 0.06	11.37 ± 0.35	6.52 ± 0.08	5.32 ± 0.07	10.71 ± 0.19
	2‐ heptanone	1180	1189	8.86 ± 0.03	4.41 ± 0.11	6.97 ± 0.06	8.57 ± 0.09	9.29 ± 0.03	1.29 ± 0.02
	2‐ethyl‐cyclopentanone				0.5 ± 0.03				
	acetoin	1298	1299	32.55 ± 0.86	22.56 ± 0.88	33.48 ± 0.48	19.74 ± 0.05	15.51 ± 0.41	19.87 ± 0.11
	Hydroxyacetone				0.25 ± 0.01				
	2‐nonanone			1.81 ± 0.09	0.9 ± 0.03	0.94 ± 0.01	2.04 ± 0.07	2.48 ± 0.04	5.14 ± 0.03
	2‐methyloxolan‐3‐one							0.56 ± 0.02	
	4‐cyclopentene‐1, 3‐butanedione			0.36 ± 0.02	0.24 ± 0.03		0.39 ± 0.01		
	Undecan‐2‐one			0.48 ± 0.02	0.33 ± 0.01	0.43 ± 0.02	0.5 ± 0.01	0.68 ± 0.01	1.83 ± 0.06
				52.56 ± 0.18	37.05 ± 0.15	53.19 ± 0.18	37.76 ± 0.52	33.84 ± 0.97	38.84 ± 0.08
Alcohols	Ethanol							1.87 ± 0.02	
	3‐methylbut‐3‐en‐1‐ol							0.71 ± 0.02	
	Hexan‐1‐ol			0.32 ± 0.02				0.54 ± 0.01	
	D‐su‐O‐threonine ethyl ester			1.29 ± 0.02					
	2,3‐butanediol							4.85 ± 0.06	
	Furfuryl alcohol			10.38 ± 0.02	7.1 ± 0.2	8.97 ± 0.05	8.46 ± 0.03	7.98 ± 0.03	
				11.99 ± 0.34	7.1 ± 0.2	8.97 ± 0.05	8.46 ± 0.54	15.95 ± 0.52	
Aldehydes	Furfural			1.65 ± 0.04	1.01 ± 0.05	1.36 ± 0.04	1.22 ± 0.04	1.56 ± 0.03	
Acids	Acetic acid	1485	1425	15.78 ± 0.04	25.40 ± 0.05	15.03 ± 0.04	13.54 ± 0.05	25.32 ± 0.32	2.02 ± 0.06
	Isobutyric acid				0.33 ± 0.01	0.7 ± 0.03	1.39 ± 0.02		
	Butyric acid	1667	1652	4.00 ± 0.01	4.98 ± 0.03	4.01 ± 0.01	5.64 ± 0.05	4.00 ± 0.01	7.24 ± 0.05
	DL‐3‐methylvaleric acid							0.75 ± 0.04	
	Isovaleric acid			1.94 ± 0.05	4.77 ± 0.11	2.42 ± 0.55	2.69 ± 0.12		
	Hexanoic acid	1928	1925	6.2 ± 0.03	13.15 ± 0.13	8.98 ± 0.07	17.43 ± 0.10	10.23 ± 0.07	34.40 ± 1.87
	2‐ethylhexanoic acid			0.28 ± 0.11		0.42 ± 0.02			
	Isovaleric acid					0.43 ± 0.02			
	Butanecarboxylic acid			0.24 ± 0.01	0.43 ± 0.02		0.43 ± 0.02		0.80 ± 0.01
	Octanoic acid	2056	2084	4.29 ± 0.02	6.07 ± 0.30	4.70 ± 0.05	9.83 ± 0.04	4.70 ± 0.01	21.37 ± 0.09
	Nonanoic acid	2187	2192			0.46 ± 0.01	0.6 ± 0.02	0.09 ± 0.01	
				32.73 ± 0.04	55.13 ± 0.09	37.15 ± 0.09	51.55 ± 0.05	45.09 ± 0.07	65.84 ± 0.42
Ethers	1‐methoxy‐2‐[2‐[2‐[2‐[2‐(2‐methoxyeth‐oxy)ethoxy] ethoxy]ethoxy] ethoxy]ethane							0.26 ± 0.03	
	1‐methyl octyl ether							0.43 ± 0.04	
Esters	Ethyl 2‐hydroxy‐2‐(4‐hydroxyphenyl)‐acetate			0.48 ± 0.01					
	o‐methoxycarbonylphenolate							0.29 ± 0.06	
Polyaromatic hydroarbons	Styrene						0.77 ± 0.01		
	Anthracen‐1‐amine			0.77 ± 0.01					
	4‐phenyl‐3H‐1,3‐thiazole‐2‐thione							0.59 ± 0.08	
	2‐acetylfuran						0.31 ± 0.01	0.32 ± 0.01	
	1‐benzyl‐3‐ methyl‐ ethylene urea							0.75 ± 0.01	

Note

Values are expressed as mean ± SD. 1–5: *P. pentosaceus*, 1–7: *P. pentosaceus*, 1–19: *E. faecium*, 1–12: *L. plantarum*,1–15: *L. plantarum*, control: *L. delbrueckii* subsp. *Bulgaricus*.

The characteristic aroma of fermented milk products is extremely variable because it is strongly influenced by the enzymatic activities of indigenous and/or added microorganisms (Routray & Mishra, 2011; Tian, Shi, et al., 2019; Tian, Xu, et al., 2019). As shown in Table 2, ketones and acids were the most abundant compounds in the yogurt aromatic profiles, followed by alcohols and aldehydes. Ketones are generated from the β‐oxidation of acyl lipids and are a rich constituent of many dairy products (Matera et al., 2018; Tian, Shi, et al., 2019; Tian, Xu et al., 2019). These compounds have characteristic odors and low perception thresholds (Frank et al., 2004). The highest ketone production occurred in *Pediococcus pentosaceus* (1–5) and *Enterococcus faecium* (1–19) (52.56% and 53.19%, respectively), consistent with previous findings (Guarrasi et al., 2017). Among the identified ketones, 2,3‐butanedione and acetoin were detected at high levels for 1–5 and 1–19 (41.05% and 44.85%, respectively), and at low levels for 1–12 and 1–15, (26.26% and 20.83%, respectively) compared to 1–7 and the control (30.42% and 30.58%, respectively). The 2,3‐butanedione and acetoin are generated from pyruvate, which comes from lactose in milk and citrate metabolism (Dan et al., 2017; Pan et al., 2014). Both of these compounds impart a “buttery, creamy, vanilla” flavor to yogurt (Dan et al., 2017; Settachaimongkon et al., 2014). Many studies have demonstrated that 2,3‐butanedione, a diketone, can readily be converted to acetoin by the enzyme diacetyl reductase (Carballo et al., 1991; Dan et al., 2017; Rattray et al., 2003). The flavor characteristics of 2,3‐butanedione and acetoin are similar, and in combination, they contribute to the “buttery” flavor of fermented milk products; they significantly contribute to their buttery and creamy aromas (Dan et al., 2017; Nieto‐Arribas et al., 2011). Other ketones, such as 2‐heptanone, 2‐nonanone, and 2‐methyl‐n‐nonone were also detected. The relative abundance (%) of 2‐heptanone for the five isolates was higher than that of the control. 2‐heptanone, 2‐nonanone, and 2‐methyl‐n‐nonone contribute to herbaceous, fruity, floral, and creamy yogurt odors and are considered favorable for flavoring milk products (Bezerra et al., 2017).

Acids are aromatic compounds that have important effects on yogurt flavor (Bezerra et al., 2017; Routray & Mishra, 2011). Acids accounted for 32.73% (1–5), 55.13% (1–7), 37.15% (1–19), 51.55% (1–12), 45.09% (1–15), and 65.84% (control) of the total compounds. Acids can be generated through either lipolysis or glycolysis, but they are mainly generated through lactose metabolism (Hayaloglu et al., 2013). Acids are also used in the formation of other aromatic compounds such as ketones and alcohols (Delgado et al., 2010). Acetic acid was the major acid detected in all five isolates (>13%). Acetic acid produces a vinegar flavor and is associated with the slight tart taste of dairy products (Tian, Shi, et al., 2019; Tian, Xu, et al., 2019). However, the control generated a higher relative abundance (%) of hexanoic (34.40%) and octanoic (21.37%) acid than the isolates, with the exception of 1–12 (*Lactobacillus plantarum*); the relative abundances (%) of hexanoic and octanoic acid were 17.42% and 9.83%, respectively, which were slightly higher than those of the other four isolates. Butyric acid was also detected in all the yogurt samples. Hexanoic acid, which originates from lipolysis, produces a light cream flavor. Butyric and octanoic acids contribute to the characteristic flavor of cheeses (Delgado et al., 2010).

The classes of alcohols and aldehydes followed those of ketones and acids in terms of the relative abundance (%) of volatile compounds for the five isolates, and were not detected in the control strain yogurt. Furfuryl alcohol was the major alcohol present. Although alcohols have a limited influence on flavor due to their high sensory thresholds, they represent an index of the fermentation process (Langler et al., 1967). Furfural was the only aldehyde detected in the yogurts.

The abundant and unique volatile compounds present in the fermented yogurts may be one reason for the singular flavor of traditional Kazakh fermented dairy products.

### 3.5 PCA of volatile compound profiles of yogurts prepared with different strains

**FIGURE 3:**
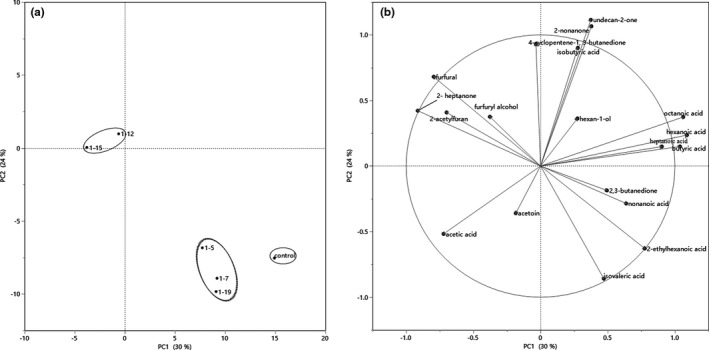
Principal component analysis (PCA) on flavor compounds. (a) PCA plots from separation of yogurts prepared with the six strains, and (b) Distribution of volatile compounds in yogurt prepared with the six strains based on content

PCA of volatile compounds of yogurts prepared with different starter cultures based on the content was conducted. In Figure 3a, the scatter plot for the two first principal components (PC1 and PC2) represents the differences among the samples fermented by the six strains. The corresponding loading plot was illustrated in Figure 3b, which represent the relative importance of each volatile compound and the relationships between volatile compounds and samples (Zhang et al., 2020). PC1 and PC2 explained 30% and 24% of the total variation, respectively.

Samples prepared with the six strains could be clustered into three groups. The first group (1–12 and 1–15) was located in the negative region of PC1 and the positive region of PC2. 1–5, 1–7, and 1–19 were clustered into the second group. The third group only contained the control sample. This distribution of PCA also suggested the flavor uniqueness of the traditional Kazakh fermented dairy products.

As shown in Figure 3b, five flavor compounds (2‐heptanone, furfural, furfural alcohol, and other two polyaromatic hydrocarbons) were located in the negative region of PC1 and the positive region of PC2. 2‐Heptanone, furfural, and furfural alcohol are typical compounds detected in fermented milk products (Cheng, 2010). Although 1–5, 1–7, and 1–19 were clustered into same group, they were located in the positive region of PC1 and the negative region of PC2, as the control was. There were total of four flavor compounds in this region, including three acids and one ketone. Those compounds were also found in yogurt products (Cheng, 2010).

### 3.6 Sensory qualities

Sensory evaluation results for the five yogurt samples are shown in Table 3. Yogurts made with strains 1–12 and 1–15 received positive overall sensory quality scores for a smooth yogurt gel appearance and relatively satisfactory fermented flavor. This evaluation may be a result of their good acidification and coagulation ability, condensed and textural integrity, and diverse and unique flavors.

**TABLE 3:** Sensory evaluation of yogurt samples

Species	Strain	Appearance	Flavor	Taste	Overall quality
*Pediococcus pentosaceus*	1–5	8.03 ± 0.48^b^	5.44 ± 0.31^b^	6.30 ± 0.41^b^	6.59 ± 0.22^b^
*Pediococcus pentosaceus*	1–7	8.64 ± 0.27^c^	5.66 ± 0.32^b^	7.48 ± 0.24^c^	7.26 ± 0.18^c^
*Enterococcus faecium*	1–19	6.16 ± 0.39^a^	4.71 ± 0.32 ^a^	5.51 ± 0.41^a^	5.46 ± 0.17^a^
*Lactobacillus plantarum*	1–12	9.45 ± 0.25^e^	7.73 ± 0.33^d^	8.51 ± 0.23^e^	8.56 ± 0.16^e^
*Lactobacillus plantarum*	1–15	9.04 ± 0.24^d^	6.94 ± 0.26^c^	7.85 ± 0.37^d^	7.94 ± 0.20^d^
*Lactobacillus delbrueckii* subsp. *Bulgaricus*	Control strain	9.16 ± 0.19^d^	8.91 ± 0.38^e^	9.10 ± 0.39^f^	9.06 ± 0.22^f^

Note

Values are expressed as mean ± SD. Different letters within the same column indicate significant differences (*p < .05).

These results suggest that strains 1–12 and 1–15 can be used as adjunct cultures with a control strain, or other commercial strains, for manufacturing fermented dairy products with a unique flavor (Crow et al., 2001; Leroy & De Vuyst, 2004).

## 4 CONCLUSION

Our results showed that the yogurts fermented by the five LAB that were isolated from fermented milk products in Xinjiang, China had the unique flavor and sensory qualities. Two isolates, strains 1–12 (*Lactobacillus plantarum*) and 1–15 (*Lactobacillus plantarum*), might be practically applied as adjunct cultures for the production of fermented milk products because of their desirable acidifying capacity, condensed texture, and pleasing flavor qualities. Creating starters by mixing these native strains with commercial LAB should be considered to develop distinctive, traditional nomadic fermented milk products to satisfy consumer demand and increase market acceptability.

## CONFLICT OF INTEREST

The authors declare no conflict of interest.

## AUTHOR CONTRIBUTION

**Hui Li:** Data curation (lead); Project administration (lead); Writing – review & editing (equal). **Jiaxing Gao:** Formal analysis (equal); Validation (equal). **Wenbo Chen:** Data curation (equal); Writing – original draft (equal); Writing – review & editing (lead). **Chengjing Qiang:** Data curation (supporting). **Yong Wang:** Writing – review & editing (supporting). **Jing Wang:** Methodology (equal); Supervision (equal). **Lishui Chen:** Supervision (supporting).

## ETHICAL APPROVAL

This study does not involve any human or animal testing.

## Data Availability

The data that support the findings of this study are available from the corresponding author upon reasonable request.
